# Patient-specific cranio-spinal compliance distribution using lumped-parameter model: its relation with ICP over a wide age range

**DOI:** 10.1186/s12987-018-0115-4

**Published:** 2018-11-15

**Authors:** Ritambhar Burman, Noam Alperin, Sang H. Lee, Brigit Ertl-Wagner

**Affiliations:** 1 Biomedical Engineering Department, University of Miami, Coral Gables, FL 33146 USA; 2 Radiology Department, University of Miami, Miami, FL 33136 USA; 3 Department of Radiology, Ludwig-Maximilians University, 80539 Munich, Germany

**Keywords:** Cerebrospinal fluid dynamics, Cranio-spinal compliance distribution, Intracranial pressure

## Abstract

**Background:**

The distribution of cranio-spinal compliance (CSC) in the brain and spinal cord is a fundamental question, as it would determine the overall role of the compartments in modulating ICP in healthy and diseased states. Invasive methods for measurement of CSC using infusion-based techniques provide overall CSC estimate, but not the individual sub-compartmental contribution. Additionally, the outcome of the infusion-based method depends on the infusion site and dynamics. This article presents a method to determine compliance distribution between the cranium and spinal canal non-invasively using data obtained from patients. We hypothesize that this CSC distribution is indicative of the ICP.

**Methods:**

We propose a lumped-parameter model representing the hydro and hemodynamics of the cranio-spinal system. The input and output to the model are phase-contrast MRI derived volumetric transcranial blood flow measured in vivo, and CSF flow at the spinal cervical level, respectively. The novelty of the method lies in the model mathematics that predicts CSC distribution (that obeys the physical laws) from the system dc gain of the discrete-domain transfer function. 104 healthy individuals (48 males, 56 females, age 25.4 ± 14.9 years, range 3–60 years) without any history of neurological diseases, were used in the study. Non-invasive MR assisted estimate of ICP was calculated and compared with the cranial compliance to prove our hypothesis.

**Results:**

A significant negative correlation was found between model-predicted cranial contribution to CSC and MR-ICP. The spinal canal provided majority of the compliance in all the age groups up to 40 years. However, no single sub-compartment provided majority of the compliance in 41–60 years age group. The cranial contribution to CSC and MR-ICP were significantly correlated with age, with gender not affecting the compliance distribution. Spinal contribution to CSC significantly positively correlated with CSF stroke volume.

**Conclusions:**

This paper describes MRI-based non-invasive way to determine the cranio-spinal compliance distribution in the brain and spinal canal sub-compartments. The proposed mathematics makes the model always stable and within the physiological range. The model-derived cranial compliance was strongly negatively correlated to non-invasive MR-ICP data from 104 patients, indicating that compliance distribution plays a major role in modulating ICP.

## Background

Compliance of a distensible chamber is defined as the ratio of the change in volume and the corresponding change in pressure. A compartment that can accommodate additional volume without a large increase in pressure has large compliance. The brain and the spinal cord are contained within the cranium and the spinal canal, respectively. The cranio-spinal (CS) compartment is filled with the cerebrospinal fluid (CSF) and sealed by the thick dura mater. The individual compliances of the cranium and the spinal canal sub-compartments add up to determine the overall compliance of the CS system [1]. This CS compliance (CSC) governs the relationship between intracranial fluid volume and the intracranial pressure (ICP).

There is a disagreement among investigators regarding which sub-compartment, the cranial or the spinal canal, contributes more to the overall CSC in the supine posture. This is a fundamental question, as it would determine the overall role of the compartments in modulation of ICP in the healthy and diseased states. This overall CSC and relative contributions of each sub-compartment changes with a change in body posture [2]. By combining MRI CSF flow measurement and infusion techniques, Wahlin et al. [3] assumed a constant venous outflow, and concluded that the cranial compartment provides nearly two-thirds of the overall CSC. A follow-up publication by Tain et al. [4] showed that when venous drainage dynamics are accounted for, the compliance of the spinal canal dominates the overall cranio-spinal compliance and hydrodynamics. A larger spinal compliance is consistent with fact that the dura mater in the spinal canal, particularly in the lumbar region and spinal sac, is less confined by bony structures than the cranial dura matter in the cranium and upper spine.

Infusion-based methods to calculate CSC have significant limitations. In addition to risks of intracranial infection [5], the measured compliances depend on the location and dynamics of the infusion [3]. The infused amount is often large in order to overwhelm the pulsatility of CSF pressure waves [6], thereby often changing the initial state of the system. The infusion methods also do not provide the relative contributions of the cranium and spinal canal to the overall CSC.

Both generic and subject-specific lumped parameter models have been proposed to assess the CSC distribution. Gehlen et al. developed a generic lumped-parameter biomechanical model of the CSF and cardiovascular system [7] that uses arterial blood inflow to explain the hydrodynamic physiology in supine and upright position. The model assumed a lower spinal compliance contribution (35%) in supine position and showed that it further reduced in upright posture. This generic model assumes literature values of physiological parameters like elastance index, pressure offset and exponential parameter of Marmarou model [8] along with relative spinal compliance ratio for the model mechanical components to try to explain data obtained from individual subjects. Yallapragada [9] and later Tain et al. [4] used a subject-specific lumped-parameter model based on a bond-graph representation of the CS system [10], with the MRI derived net transcranial blood flow as input and the cranio-spinal CSF flow as output. The model not only predicted a higher spinal canal compliance in healthy individuals (78%), but also showed that spinal compliance contribution is lower in idiopathic intracranial hypertension patients (60%) than in control and therefore the IIH patients have a lower buffer for increased ICP. Recently, Atsumi et al. [11] modelled the bilateral carotid and vertebral arteries, and CSF flows by a transformer-coupled electrical circuit, to calculate the brain compliance index.

In this paper, we propose a new mathematical approach for the previously developed subject-specific lumped-parameter CS model of Tain et al. [4] to compute the CSC distribution in the cranium and the spinal canal. The previous model mathematics required conversion of data from discrete to continuous domain, a conversion that is not unique. The previous model also did not account for a physically viable solution where compliances are positive and have real values. The discrete transfer function predicts a set of CSC distributions (solutions for the model) by searching within a set of responses to the input parameters that yield a stable system. Each steady state response to a step function is equivalent to the dc gain of the system. The final spinal canal to cranio-spinal compliance ratio is chosen from the compliance histogram, which always provides a stable and physically realizable model, with a percentage contribution that is a real number between 0 and 100%. The cranio-spinal model utilizes the momentary transcranial blood flow (arterial minus venous) as input to predict the system transfer function that best matches the CSF flow into the spinal canal, and derives the cranio-spinal compliance distribution in the process. We hypothesize that CSC distribution is related to the cranial CSF pressure or ICP. We evaluated the relationship of the CS system to a previously developed MRI-derived ICP (MRICP) using a data from large cohort of healthy subjects over a wide age range.

## Methods

### Subjects

Following institutional review board approval, written informed consent was obtained from all subjects. In case of children, informed consent was obtained from their parents. Data from 104 out of 129 healthy individuals (48 males, 56 females, age 25.4 ± 14.9 years, range 3–60 years) were used in the study. The study population included 17 subjects in age range 3–10 years, 31 subjects in age range 11–20 years, 36 in age range 21–40 years and 20 in 41–60 age group. Seventeen cases were excluded because of data inconsistency between the arterial and venous flow where venous outflow preceded arterial inflow. This suggests active venous drainage, which the current model does not account for. Seven additional cases were excluded due to poor image quality due to subject motion during the scan. All subjects were without any history of neurological diseases, determined by self-reported and/or assessed by means of conventional MR imaging.

### MR imaging acquisition

MRI scans were acquired using a 3T scanner (Magnetom Verio; Siemens Healthcare, Erlangen, Germany) with subjects in supine position, with legs slightly elevated to improve comfort. An ECG-gated high velocity encoding cine phase-contrast scan was used to measure the arterial inflow and venous outflow to and from the cranium, with the following parameters: VENC = 70–90 cm/s, FOV = 14 × 11.4 cm, slice thickness = 6 mm, flip angle = 20°, TR/TE = 40/4.05 ms, acquisition matrix = 256 × 143, and 32 cardiac phases. A second ECG-gated low velocity encoding cine phase-contrast scan was used to measure the CSF flow at the cranio-cervical region, with the following parameters: VENC = 7–9 cm/s, FOV = 14 × 11.4 cm, slice thickness = 6 mm, flip angle = 20°, TR/TE = 53.7/7.48 ms, acquisition matrix = 256 × 143, and 32 cardiac phases. MR scan time per cine sequence was about 1.5 to 2 min (specific scan time is heartrate dependent). One average and two views per segment were used to keep acquisition time short. Imaging planes to measure blood and CSF flow were placed at the dens axis perpendicular to internal carotid and vertebral arteries, and at the mid C2 level where the spinal walls are parallel, respectively.

### MR-ICP

The calculation of non-invasive ICP with the help of phase-contrast MRI (MR-ICP) has been described previously [12]. Briefly, basis of the method is the mono-exponential relationship between volume and pressure, which makes the pressure inversely related to compliance. The compliance is defined as the ratio of intracranial volume and pressure changes during the cardiac cycle, obtained from the difference in transcranial blood and CSF volumetric flow rates, and change in CSF pressure gradient, using Navier–Stokes equation.

### Cranio-spinal lumped-parameter model

**Fig. 1:**
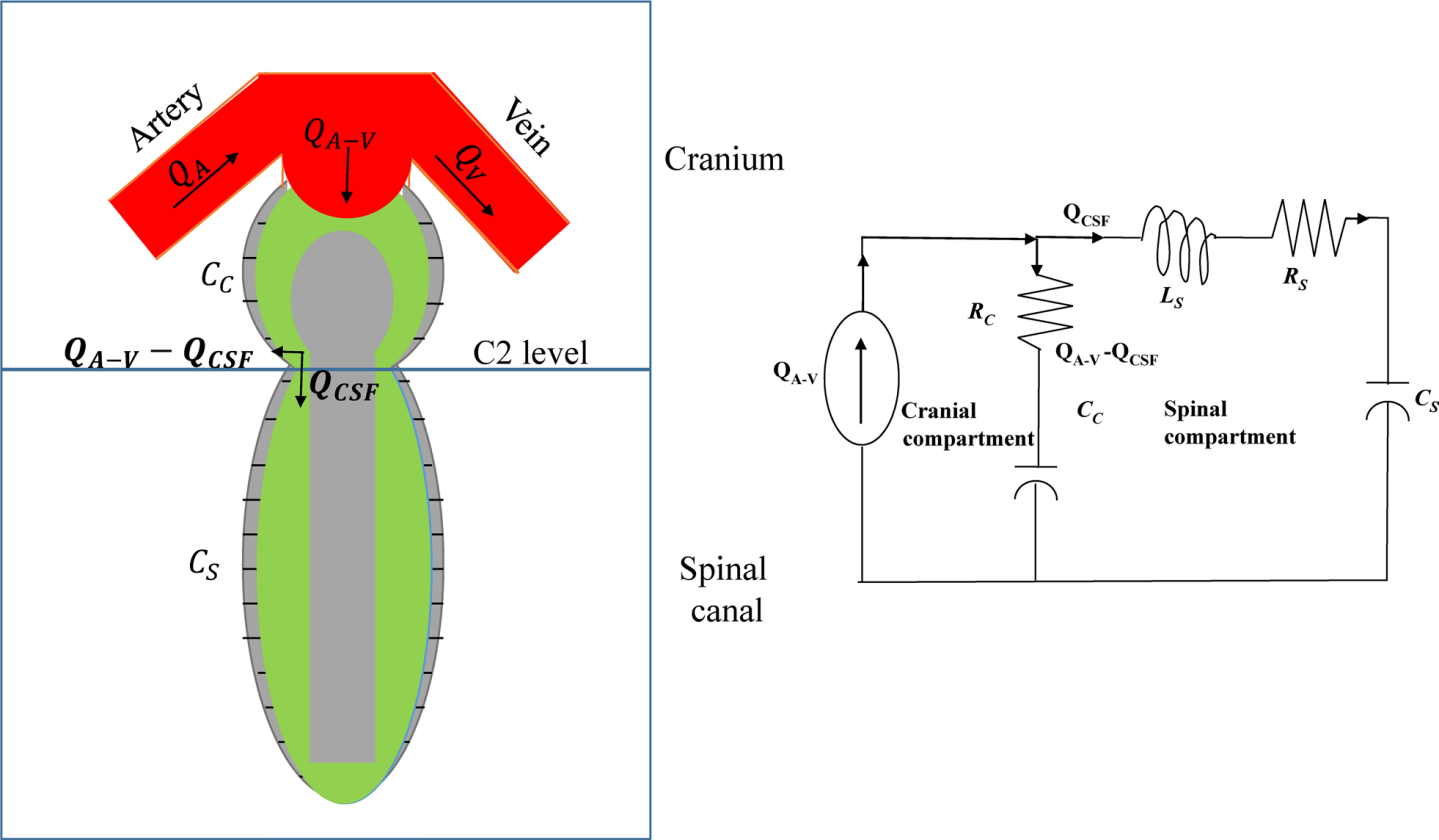
Cranio-spinal model and its electrical analogous circuit. The cranio-spinal model is divided into two compartments, cranium and spinal canal. The MR derived net transcranial blood flow *Q*_*A*−*V*_ and CSF flow *Q*_*CSF*_ are used as input and output to the model, respectively. The lumped mechanical dampers or flow resistances in the cranial and spinal compartment are denoted by *R*_*C*_ and *R*_*S*_ respectively. The compliances of the cranium and spinal canal are denoted by *C*_*C*_ and *C*_*S*_ respectively. The inertial component of the CSF flow from the cranium into the spinal canal is denoted by *L*_*S*_

The lumped-parameter model of the CS system [4] is used to determine the compliance distribution between the cranium and spinal canal. The model and its electrical equivalent are shown in Fig. 1. With each heartbeat, intracranial blood volume increases during systole. The temporary increase in the net intracranial blood volume, i.e. the difference between arterial inflow and venous outflow drives the CSF to the spinal canal. The MR derived net transcranial blood flow *Q*_*A*−*V*_ and CSF flow *Q*_*CSF*_ are used as input and output to the model, respectively. Volumetric flow rate through blood and CSF lumens were obtained by PUBS method [13] which utilize velocity dynamic information to differentiate lumen voxels from background. Arterial inflow and venous outflow rates are obtained by summing the flow velocities inside the respective lumens (left and right internal carotid arteries, and left and right vertebral arteries, and left and right internal jugular veins and secondary venous pathways for veins). The lumped mechanical dampers or flow resistances in the cranial and spinal compartment are denoted by *R*_*C*_ and *R*_*S*_ respectively. The compliances of the cranium and spinal canal are denoted by *C*_*C*_ and *C*_*S*_ respectively. The inertial component of the CSF flow from the cranium into the spinal canal is denoted by *L*_*S*_. The frequency response of the transfer function of the system, *H*(*s*) in the Laplace domain is given by Eq. (1), where *s* denotes the Laplace variable.

1 $$H\left( s \right) = \frac{{Q_{CSF} \left( {\text{s}} \right)}}{{{\text{Q}}_{{{\text{A}} - {\text{V}}}} \left( {\text{s}} \right)}} = \frac{{s\frac{{R_{C} }}{{L_{S} }} + \frac{1}{{C_{C} L_{S} }}}}{{s^{2} + s\frac{{R_{C} + R_{S} }}{{L_{S} }} + \frac{{\frac{1}{{C_{c} }} + \frac{1}{{C_{S} }}}}{{L_{S} }}}}$$

### Derivation of cranial and spinal compliance distribution from transfer function

The phase contrast MRI provides 32 discrete-time samples of *Q*_*CSF*_ and *Q*_*A*−*V*_ per cardiac cycle. Discrete to continuous-time domain conversion does not provide a unique solution as some information may be lost while sampling the transcranial flow and CSF flow from continuous to the discrete time domain [14]. Transfer function *H*(*s*) however is only valid in the continuous-time domain system. The structure of *H*(*s*) lets us calculate the compliance ratio without having to calculate the *R*_*C*_, *R*_*S*_, *L*_*S*_, *C*_*C*_ and *C*_*S*_ individually. The zero frequency gain or dc gain of the system, obtained by substituting *s* = 0 in Eq. (1), gives the spinal compliance to total CSC, *C*_*S*_/(*C*_*C*_ + *C*_*S*_). This dc gain is the amplitude ratio of the system steady state response to a step input. The discrete-time domain transfer function *H*(*z*) is given by Eq. (2), where z is the z-transform variable for discrete model. Continuous-time transfer function and its discrete-time domain equivalent have the same form and same dc gain in pole-zero matched method [15]. Thus *H*(*z*) is represented by the quartet $\left( {p_{1} , p_{2} , q_{1} , q_{2} } \right)$, which are real numbers which makes the form of *H*(*z*) correspond to *H*(*s*). The corresponding dc gain, obtained by substituting *z* = 1 in *H*(*z*), estimates the compliance distribution in the cranium and the spinal canal as it is equivalent to H (s = 0).

2 $$H\left( z \right) = \frac{{Q_{CSF} \left( z \right)}}{{Q_{A - V} \left( z \right)}} = \frac{{p_{1} z + p_{2} }}{{z^{2} + q_{1} z + q_{2} }}$$

### Procedure to estimate accuracy and dc gain of second order stable model

Roots *Z*_*p*_ of the second order transfer function *H*(*z*) in Eq. (2) follow the form given by Eq. (3). The discrete system, and subsequently its continuous counterpart, are stable if both poles of *H*(*z*), *Z*_*p*_, lie within unit circle from the origin, given by (4). The modulus of sum ($\left| {q_{1} } \right|$) and product ($\left| {q_{2} } \right|$) of poles of such a second order transfer function is less than 2 and 1 respectively, given by Eq. (5) and Eq. (6) respectively. The fourth constraint, given by Eq. (7), is real domain of coefficients *q*_1_ and *q*_2_ due to presence of complex conjugate poles in second order transfer function. While Eq. (3) and Eq. (4) are sufficient, Eq. (5–7) are necessary, but not sufficient to achieve a stable system.

3 $$Z_{p} = \frac{{ - q_{1} \pm \sqrt {q_{1}^{2} - 4q_{2} } }}{2}$$

4 $$\left| {Z_{p} } \right| < 1$$

5 $$\left| {q_{1} } \right| < 2$$

6 $$\left| {q_{2} } \right| < 1$$

7 $$q_{1} ,q_{2} \in R$$

Equations (4–7) can be used to draw the *q*_1_ − *q*_2_ mesh-grid domain in steps of 0.001 along each axis, which always gives a stable second order system. For a fixed (*q*_1_, *q*_2_) pair and known values of input *Q*_*A*−*V*_(*n*) and output *Q*_*CSF*_(*n*), ordinary least squared estimate can be used to estimate (*p*_1_, *p*_2_) in Eq. (8) that minimizes the right-hand side for all 32 frames of the cardiac cycle. For a given quartet (*q*_1_, *q*_2_, *p*_1_, *p*_2_), $\hat{Q}_{CSF} \left( n \right)$ can be estimated for all 32 frames of the cardiac cycle using Eq. (2). Each quartet in CS model gives a different dc gain and hence a different compliance distribution, obtained by substituting *z* = 1 in Eq. (3). The power of the model is how well the predicted CSF flow matches the observed flow, and is represented by Nash–Sutcliffe coefficient *E* [16], given by Eq. (9). *E* can take values between −∞ and 1, with better model accuracy indicated by a larger *E*.

8 $$Q_{CSF} \left( n \right) + q_{1} Q_{CSF} \left( {n - 1} \right) + q_{2} Q_{CSF} \left( {n - 2} \right) = p_{1} Q_{A - V} \left( {n - 1} \right) + p_{2} Q_{A - V} \left( {n - 2} \right)$$

9 $${\text{E}} = 1 - \frac{{\mathop \sum \nolimits_{n = 1}^{32} \left( {Q_{CSF} \left( {\text{n}} \right) - \hat{Q}_{CSF} \left( n \right)} \right)^{2} }}{{\mathop \sum \nolimits_{n = 1}^{32} \left( {Q_{CSF} \left( {\text{n}} \right) - \bar{Q}_{CSF} \left( {\text{n}} \right)} \right)^{2} }}$$

where $\bar{Q}_{CSF} \left( n \right)$ denotes the mean value of the *Q*_*CSF*_ over 32 frames of the cardiac cycle.

### Calculation of compliance distribution in the CS system

Among all the stable systems with $E > 0.7,\,C_{S} /\left( {C_{C} + C_{S} } \right)$ is constrained to be in a range between 0 and 100% in steps of 1%, thereby making the model physically realizable. This provides a histogram of solutions. The final solution *C*_*S*_/(*C*_*C*_ + *C*_*S*_) is the maximum of the histogram, representing the most commonly occurring compliance ratio. The cranial compliance counterpart is obtained by subtracting the spinal contribution from 100%.

### Statistical analysis

Statistical analyses were performed using Excel (Windows Version 2016). All data were presented as mean ± standard deviation or as median and interquartile range. Univariate analyses were performed for analyzing the effect of gender and age on cranial contribution to CSC. Multi-variate analysis was used to analyze the effect of age and gender on cranial contribution to CSC. Ordinary least squared estimate was used wherever relevant and smoothing spline was used in some graphs for illustrative purposes. Pearson correlation coefficient was used to measure the strength of linear regressions. A two-sided Student’s t-test was used throughout the analysis, and a p of < 0.05 was considered as statistically significant.

## Results

### Overview

**Fig. 2:**
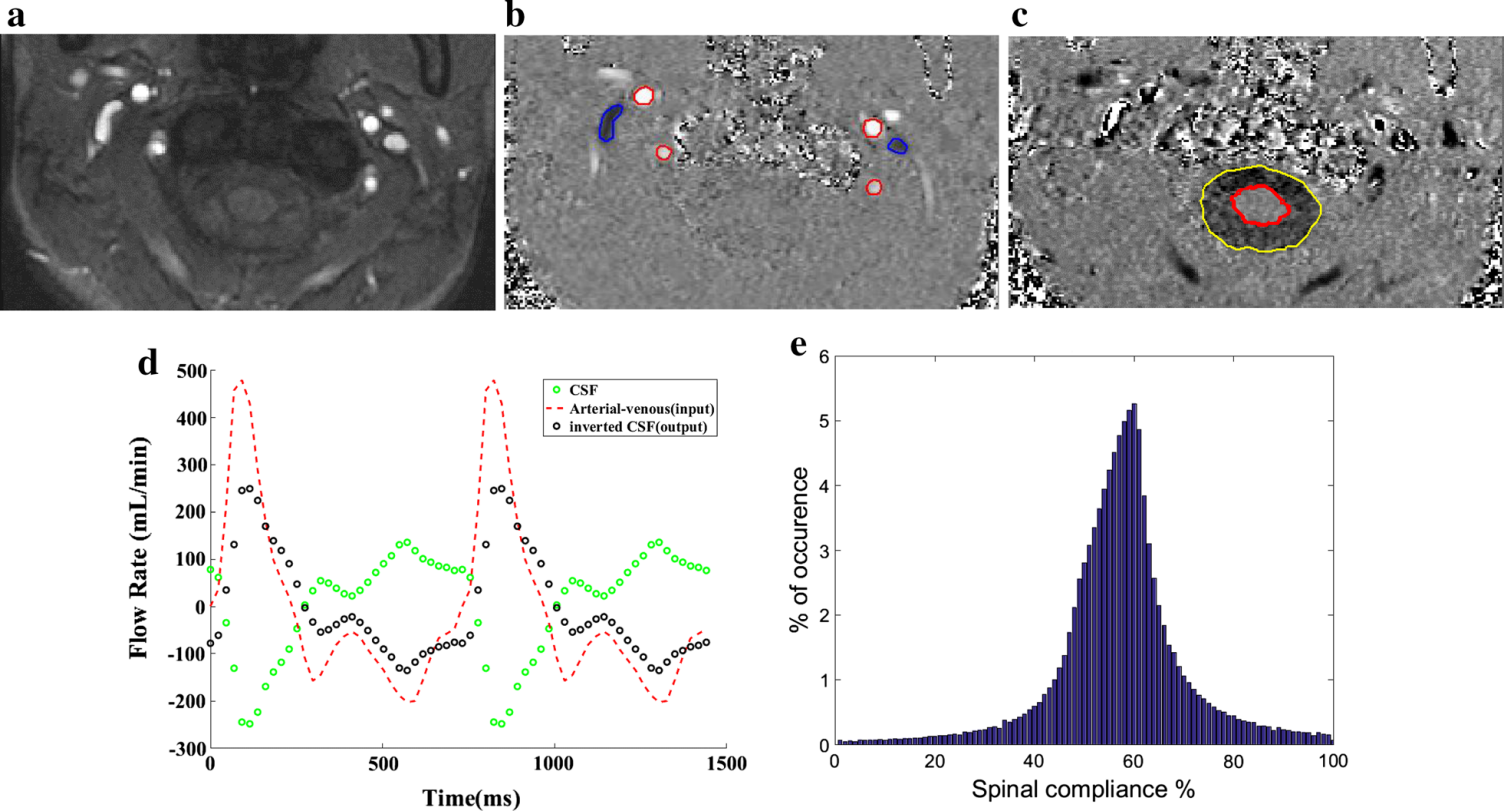
Example of determination of cranial contribution to cranio-spinal compliance from sample input and output waveforms. **a** Flow compensated magnitude image showing bright signal from blood vessels. **b** High-velocity encoding images used for measurements of arterial inflow and venous outflow. **c** Low-velocity encoding images used for measurements of CSF flow. **d** Phase contrast MRI derived cardiac cycle of *Q*_*A*−*V*_ (red) and *Q*_*CSF*_ (green) in an 21-year-old healthy female subject is plotted. *Q*_*A*−*V*_ is used as input to the model, which predicts the inverted *Q*_*CSF*_ (black) waveform. **e** Histogram corresponding to model-derived spinal contribution to cranio-spinal compliance, *C*_*S*_/(*C*_*C*_ + *C*_*S*_), is plotted for all the model parameters that give *E* > 0.7. The final spinal contribution to CSC is chosen from the mode of the histogram (60%)

An example of cine images which include the magnitude images and the two phase images with a high and low VENC for a 21 year-old healthy female subject is shown in Fig. 2a–c. MRI derived *Q*_*A*−*V*_ and *Q*_*CSF*_ flow waveforms are shown in Fig. 2d. In Fig. 2e, the solutions histogram is plotted for all quartets (*q*_1_, *q*_2_, *p*_1_, *p*_2_) which gives *E* > 0.7, with the vertical axis indicating the frequency of occurrence of the 100 possible compliance percentages (along the horizontal axis). The spinal compliance to CSC ratio is 60% in this case.

### Compliance distribution in the CS system and its relation with ICP

**Fig. 3:**
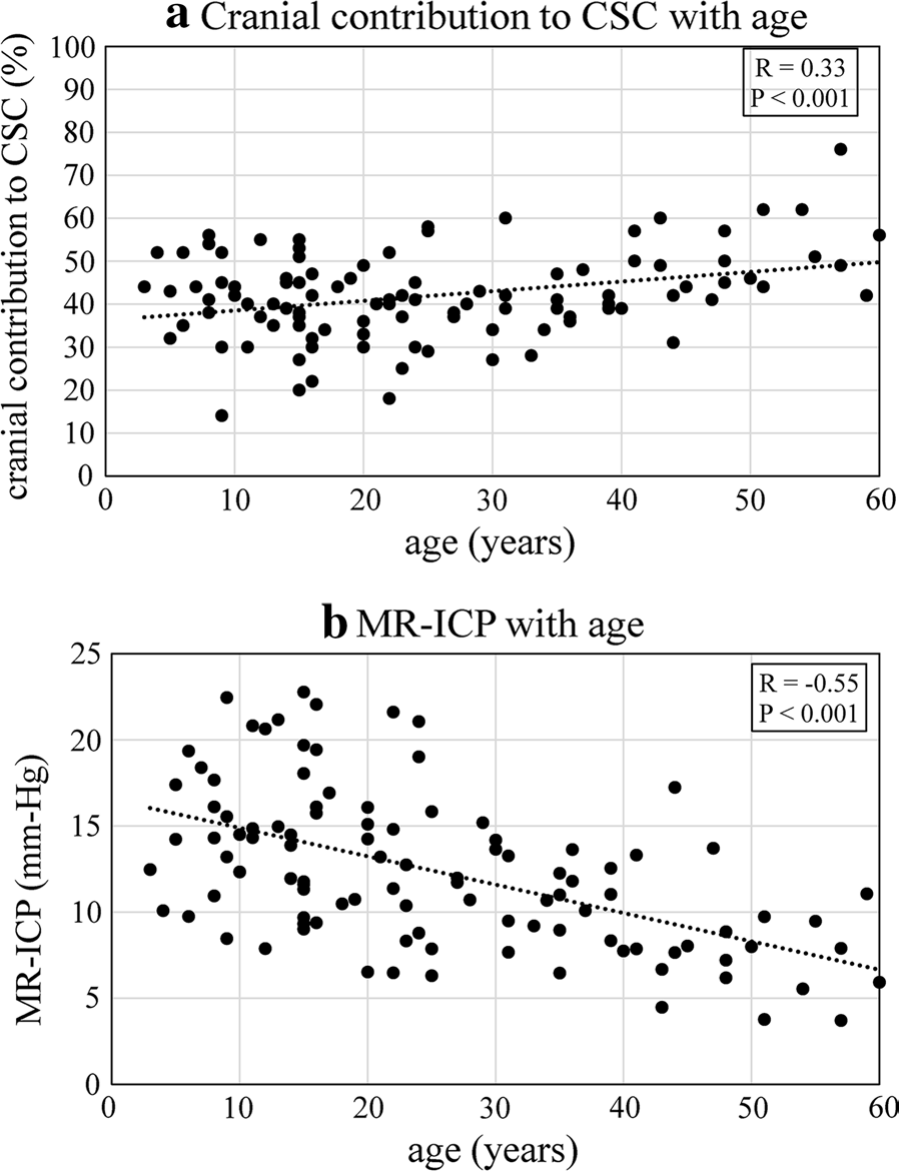
Effect of age on cranial contribution to cranio-spinal compliance distribution and MR-ICP. **a** The plot shows significant positive correlation (p < 0.001, R = 0.33) between cranial contribution to CSC and age. **b** The plot shows significant negative correlation (p < 0.001, R = − 0.55) between non-invasively determined MR-ICP and age

**Fig. 4:**
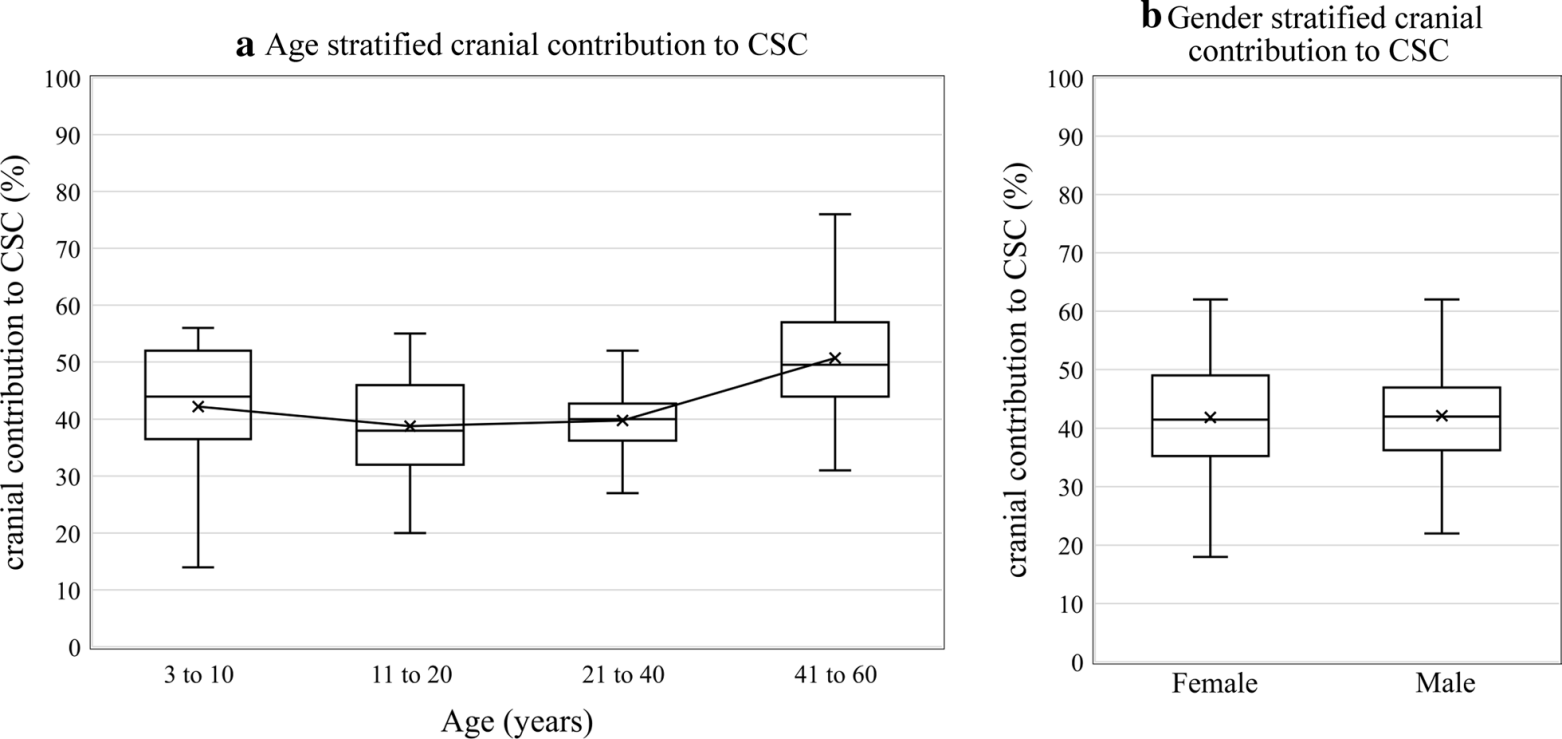
Effect of age and gender on cranial contribution to cranio-spinal compliance distribution. **a** Boxplot of cranial contribution to CSC and stratified age shows the cranial compliance contribution decreases slightly in the younger population before increasing in the 20+ population. **b** Boxplot of cranial contribution to CSC does not vary significantly with gender (p = 0.88)

Linear regression of cranial contribution to CSC and MR-ICP with age (Fig. 3a, b) showed significant positive (p < 0.001, R = 0.33) and negative correlations (p < 0.001, R = − 0.55), respectively. The cranial contribution to CSC in 104 subjects, stratified by age and sex, respectively are shown in Fig. 4a, b. The age was stratified into 3–10 years, 11–20 years, 21–40 years and 41–60 years. When analyzed over different age groups (Fig. 4a), median cranial contribution to CSC was found to be the highest in the older population of 41–60 years (median = 49.5%, IQR 44%–57%), with the lowest median cranial contribution to CSC of 38% occurring in the age range of 11–20 years (IQR 32%–46%). Spinal compliance was significantly greater than the cranial compliance for the age groups 3–10 years (p = 0.008), 11–20 years (p < 0.001), and 21–40 years (p < 0.001). However, no significant difference was found in the compliance contribution of the two compartments in the older population of age 41–60 years (p = 0.75).

The median cranial compliance was equal in both males (median = 42%, IQR 36%–47%) and females (median = 42%, IQR 35%–49%) (Fig. 4b) with no significant statistical difference (p = 0.88). Multivariate regression analysis of cranial contribution to CSC with both age and gender, showed that only age has a significant influence on the compliance distribution (p for age < 0.001, p for gender = 0.54).

**Fig. 5:**
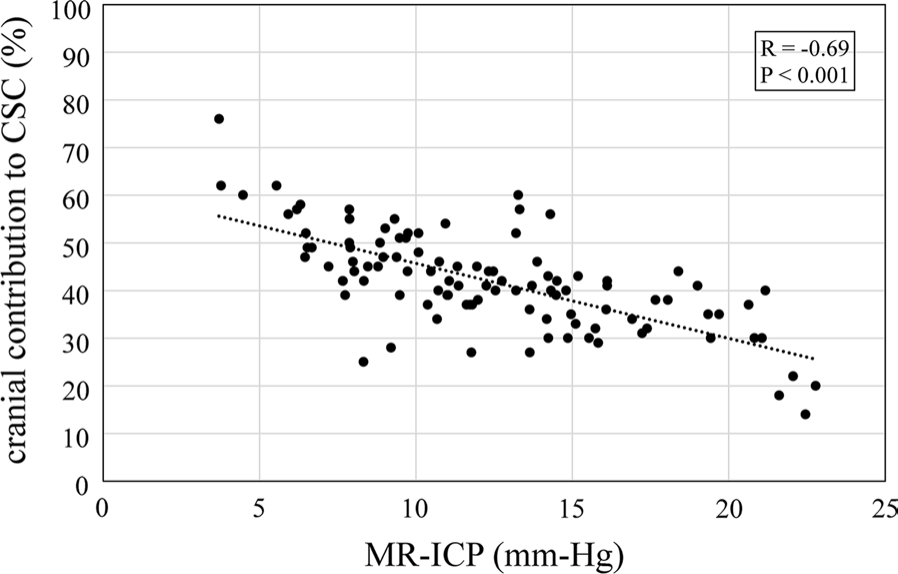
Relationship between cranial contribution to cranio-spinal compliance distribution and MR-ICP. Significant negative correlation is shown between cranial contribution to CSC on MR-ICP (p < 0.001, R = − 0.69)

Scatterplot of model-derived cranial contribution to CSC and MR-ICP is shown in Fig. 5. Least squared regression showed a significant negative correlation of the cranial compliance contribution with the MR-ICP (p < 0.001, R = − 0.69).

### Spinal compliance, CSF stroke volume and total cranial blood flow

**Fig. 6:**
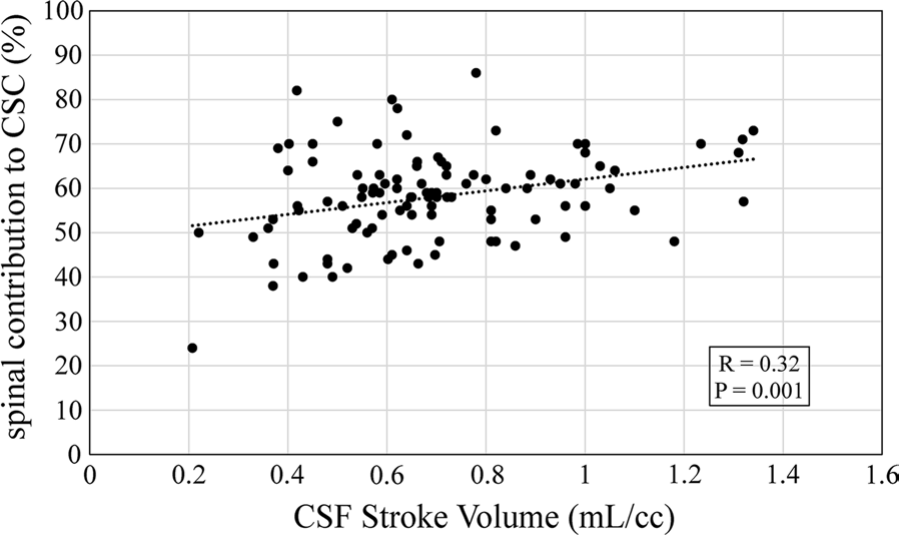
Effect of CSF stroke volume on spinal contribution to cranio-spinal compliance distribution. The plot shows a significant positive regression of spinal contribution to CSC on CSF stroke volume (p = 0.001, R = 0.32)

**Fig. 7:**
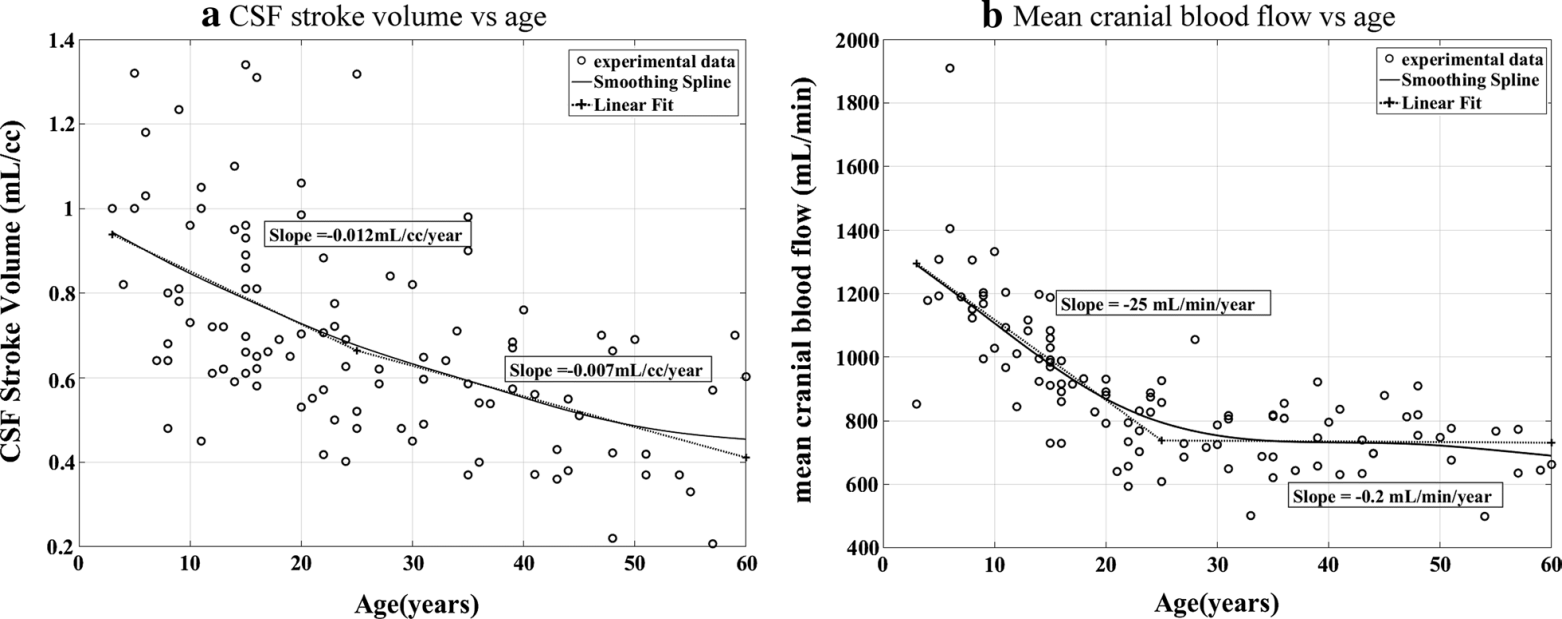
Effect of age on CSF stroke volume and mean cranial blood flow. **a** Significant negative correlation is shown between CSF stroke volume (mL per cardiac cycle) at C2 level and age (Spline fit p < 0.001, R = − 0.74). **b** Significant negative correlation is shown between transcranial blood flow and age (Spline fit p < 0.001, R = 0.82)

Linear regression of CS CSF stroke volume with respect to spinal compliance (Fig. 6) showed a significant positive correlation (p = 0.001, R = 0.32). The dependence of CS CSF stroke volume with Linear regressions of CSF stroke volume and mean cranial blood flow with age showed significant negative correlations (p < 0.001, R = − 0.74, Fig. 7a; p < 0.001, R = − 0.82, Fig. 7b, respectively).

### Sensitivity of the parameters

The reproducibility of the compliance contribution for a given data set can be affected by the Nash–Sutcliffe coefficient *E*. It provides a measure of how accurate the model parameters describe the output of the system when compared to its original waveform. For the cases studied, the least squared estimated best-fit case has an *E* value between 0.76 and 0.96, except one case which had an E equal to 0.69. For the compliance estimation, the value of 0.7 was empirically decided to be the threshold for *E*. To calculate the goodness of the threshold, we also recalculated the compliance contribution from each of the two models with a lower threshold of E = 0.6. The absolute difference of compliance predicted by E = 0.7 and E = 0.6 in the two models was 2.2% ± 1.7% (0%–5%), with two out of 104 cases having a compliance difference of more than 10% and excluded from this analysis.

The step-size in *q*_1_ − *q*_2_ domain is chosen to be 10^−2^ in this study. It was found that a finer step size of 10^−3^ converged the system to the same compliance value. A finer step size however is computationally inefficient.

## Discussion

### Overview

This paper describes a modular patient-specific lumped-parameter model to non-invasively determine the distribution of the total CS compliance between the cranium and the spinal canal. The measurement of CSF flow by MRI in the upper spine provided us means to separate the CS system to two sub compartments, which infusion based method are incapable. The patient-specific model utilizes the measurements of blood and CSF flows to and from the two sub-compartments. The model further demonstrates that the cranial contribution to total cranio-spinal compliance distribution is significantly negatively correlated with the non-invasively derived MR-ICP. This inverse relationship is expected and can be explained by the inverse relation of absolute ICP with compliance. The inverse relationship between ICP and compliance is due to the mono-exponential relationship between ICP and intracranial volume [8].

The model-predicted spinal compliance over total compliance is significantly higher than the cranial contribution in population of age less than 40 years. This may be attributed to the less-confined dura matter in the spinal canal relative to that in the cranium. A higher spinal compartment compliance is in accord with previous results which used invasive techniques (70% [1] and 63% [17]). Lofgren and Zwetnow [1] infused artificial CSF in the cisterna magna in six dogs after isolating the cranial and spinal compartments using a block at the C1 level, and found that the spinal section contributed to 70% of volume change. The finding of a larger spinal compliance is consistent with a much earlier report by Magnaes [17], who used bolus infusion separately into the cranium and the spinal canal in human patients with CSF block at cervical level. These results contrast with recent studies suggesting that the cranium contribution is a dominant source of CSC in supine position [3, 7, 8] accounting for almost two-thirds of the total CSC. Wahlin [3] assumed constant relative pulse pressure coefficient in the cranio-spinal compliance to quantify 65% compliance coming from the intracranial compartment in thirty-seven healthy elderly subject. Marmarou et al. [8] determined that nearly two-third of the compliance came from the cranial compartment in anaesthetized adult cats with cranio-spinal compartment isolated at c6 level.

When looked at the effect of age on the compliance distribution, our study found significantly higher contribution of spinal canal to CSC in young subjects (age less than 40 years), and non-significantly lower spinal contribution to CSC on an average in the 41–60 age group. Wahlin [3] reported 35% spinal contribution to CSC in 37 healthy adults (60–82 years of age), while unpublished data from our group showed 61.9% spinal compliance in ten healthy adults (60–79 years of age). This anomaly may be due to lack of Body Mass Index data of the subjects in [3] that makes the study group population incomparable. Body Mass Index is significantly negatively correlated with cranial compliance, which in turn is significantly positively correlated with ICP (Ritambhar Burman, Ashish Shah, Ronald Benveniste, George Jimsheleishvili, Sang Lee, David Loewenstein, Noam Alperin). Literature suggests that in obese patients, the intra-thoracic pressure is greater than normal [18], which causes a significant increase in ICP by causing a functional obstruction to cerebral venous outflow via jugular venous system [19].

In addition to the cardiac pulsation, respiration also influences the CSF dynamics [20]. The respiration effect is superimposed on the cardiac pulsation. During exhalation, the intrathoracic pressure increase results in lower venous drainage and therefore increased ICP and CSF is pushed to the spinal canal, while during inspiration, the reverse occurs [21]. The derived images from the cine scan represent an average cardiac cycle over more than a minute and therefore respiratory effect is averaged out. Only the cardiac related components are considered by the model.

### Relationship of cranial compliance contribution with ICP

The strong negative correlation between cranial compliance contribution and the non-invasively determined MR-ICP supports our hypothesis that the CS distribution plays a major role in regulating the ICP. Small increases in intracranial volume can result in exponential rise in ICP once the compensatory reserve volume is exhausted [22]. Cranium with a higher compensatory reserve (compliance) can accommodate larger capacity of fluid without an appreciable increase in ICP, which supports the strong negative correlation between the cranial contribution to CSC and MR-ICP. The cranial contribution to CSC was also negatively correlated to MR-ICP and lumbar puncture opening pressure in 10 healthy volunteers and External Ventricular Drain measurements in six brain trauma patients (Ritambhar Burman, Ashish Shah, Ronald Benveniste, George Jimsheleishvili, Sang Lee, David Loewenstein, Noam Alperin). The MR-ICP calculation is done using a biomechanical model [12], while the compliance calculation is done using a lumped parameter electrical model, which are not equivalent to each other.

### Relationship of cranial compliance contribution with CSF stroke volume

Strong linear correlation between CSF stroke volume at C2 level (the amount of CSF going back and forth between the cranium and spinal canal) and spinal contribution to CSC proves that a spinal canal chamber with higher contribution to CSC can accommodate higher volume of CSF outflow from a less compliant cranium. A less compliant cranium in turn has higher ICP to drive the CSF out of the cranium.

### Relationship of cranial compliance contribution with age

In our study, we observed a significant and continuous increase of cranial contribution to the total cranio-spinal compliance in the groups with age range of 20 years and above, while it showed a non-significant decrease in the younger cohorts. To our knowledge, this is one of the first papers to report the effect of age on the compliance distribution in the CS system. The mechanism of this compliance distribution in the cranium and spinal canal is affected by the complex response of these two CS compartments to aging. In patients of age 50 years or older, degenerative spinal stenosis [23], resulting from decrease in canal diameter and thicker ligamentum flavum, and calcification of the spinal canal dura result in lowered spinal canal compliance. On the other hand, the brain tissue becomes more rigid [24] with reduced CSF absorption [25], which in turn reduces the intracranial compliance [26, 27]. For the study population with age greater than 3 years, the net cranial blood flow and CSF stroke volume were also found to decrease with age, which support existing literature [28]. Both net cranial blood flow and CSF stroke volume however, increases during the first 2–3 years after birth [29]. For our study population, decreasing CSF stroke volume at C2 level with age is indicative of increasing CSF outflow resistance, which supports previous literature [25]. With the absolute values of both the cranial and spinal compliances decreasing due to age, it is difficult to ascertain the effect of age on cranio-spinal compliance ratio.

With cranial contribution to CSC negatively correlated to ICP and positively correlated to age, ICP is expected to correlate to age negatively and matches our findings. This phenomenon can be attributed to the decrease in mean cranial blood inflow with age. Fleischman et al. [30] found similar evidence, with enough power for a conclusive evidence in 12,118 patients. Several older studies with smaller sample size however have failed to find a significant relationship between ICP and age [31, 32].

### Compliance calculation and Nash–Sutcliffe coefficient *E*

In the proposed methodology, the compliance ratio is estimated from the dc gain of the system, which is estimated from the least squared estimate parameters. Thus, the dc gain estimated from only the best-fit least squared estimate parameters can be below zero or above one, implying compliance contribution of sub-compartments to be below 0% or greater than 100%. For the control model to be robust, the compliance of sub-compartments should be same over various parameter values as well, and should not rely on the best-fit case. Thus the compliance contribution is not only restricted to the physiological range of (0%, 100%), but also provides a better estimate from a pool of solutions with the guidance of Nash–Sutcliffe coefficient E. E helps to segregate the parameters that give a high fit from the ones that give poor fits. A low threshold of E will include a large number of cases including poor ones, which may not be a precise representative of the actual system. Again, the number of cases for a very high value of E will not be large enough to describe the system accurately. Thus, it is a trade-off problem. The 2.2% average deviation in compliance value when E was changed from 0.7 to 0.6 shows that the model is insensitive to threshold of E. The convergence to the same compliance value with different step sizes of $q_{1} - q_{2}$ grid shows that the model is very robust to the step-size as well.

CS compliance ratio cannot be calculated from the ratio of the average values of the output signal *Q*_*A*−*V*_ and input signal *Q*_*CSF*_ as both signals are periodic, that follow the Kellie-Monroe doctrine of conservation of CS and blood volumes in the brain over the whole cardiac cycle.

### Limitations

Several limitations of the study need to be considered when interpreting the data. The number of volunteers for each age range are limited. Our study population did not include enough volunteers to make a 60+ age group. The model-derived compliances are not absolute values, but percentages relative to the total cranio-spinal compliance.

Obtained values were not compared against the invasive gold standard technique. Such study can be done only when patients in whom invasive measurements are justified by standard clinical care. Furthermore, the lack of Body Mass Index information in the other studies we are comparing our study with, pose a challenge for comparison.

This study was done using a snapshot in time for a single subject, and hence a prospective study is needed to confirm the age related findings. The proposed model also cannot deal with active systems with pulsatile jugular flow having an onset before the arterial blood inflow. The model assumes that the arterial blood is pumped into the cranium pushing the venous blood of the cranium, which in turn drives the CSF out of the cranium.

## Conclusion

This paper describes MRI-based non-invasive way to determine the cranio-spinal compliance distribution in the brain and spinal canal sub-compartments. The proposed mathematics makes the model always stable and within the physical range, and is robust to step-size and Nash–Sutcliffe coefficient. The model-derived cranial compliance was strongly negatively correlated to non-invasive MR-ICP data in 104 subjects, indicating that compliance distribution plays a major role in modulating ICP. Consistent with the anatomical considerations, we found that the model-estimated spinal compliance contribution is greater than the cranial compliance in young subjects (age ≤ 40 years), but could not attribute either of the compartments with major source of CSC contribution in the older subjects (age > 40 years). The cranial contribution to CSC was positively correlated with age, particularly in the population of age > 20 years. MR-ICP was negatively correlated with age. Gender was not a significant factor for CSC distribution. Spinal canal contribution to CSC was strongly correlated to CSF stroke volume at the C2 level.

## Abbreviations

CS: cranio-spinal
CSF: cerebrospinal fluid
CSC: cranio-spinal compliance
ICP: intracranial pressure
IQR: interquartile range
MRI: magnetic resonance imaging
MR-ICP: magnetic resonance-based intracranial pressure
